# The co-occurrence of a four-headed coracobrachialis muscle, split coracoid process and tunnel for the median and musculocutaneous nerves: the potential clinical relevance of a very rare variation

**DOI:** 10.1007/s00276-020-02580-x

**Published:** 2020-09-26

**Authors:** Łukasz Olewnik, Nicol Zielinska, Piotr Karauda, Fabrice Duparc, Georgi P. Georgiev, Michał Polguj

**Affiliations:** 1 Department of Anatomical Dissection and Donation, Medical University of Lodz, Lodz, Poland; 2 Department of Normal and Clinical Anatomy, Medical University of Lodz, Lodz, Poland; 3 Laboratory of Anatomy, Faculty of Medicine, Rouen University, Mont-Saint-Aignan, France; 4 Department of Orthopaedics and Traumatology, Medical University of Sofia, Sofia, Bulgaria

**Keywords:** Anatomical variations, Coracobrachialis muscle, Median nerve, Musculocutaneus nerve, Split coracoid

## Abstract

The coracobrachialis muscle (CBM) originates from the apex of the coracoid process, in common with the short head of the biceps brachii muscle, and from the intermuscular septum. Both the proximal and distal attachment of the CBM, as well as its relationship with the musculocutaneus nerve demonstrate morphological variability, some of which can lead to many diseases. The present case study presents a new description of a complex origin type (four-headed CBM), as well as the fusion of both the short biceps brachii head, brachialis muscle and medial head of the triceps brachii. In addition, the first and second heads formed a tunnel for the musculocutaneus and median nerves. This case report has clear clinical value due to the split mature of the coracoid process, and is a significant indicator of the development of interest in this overlooked muscle.

## Introduction

The flexor compartment of the arm contains the biceps brachii, brachialis and coracobrachialis (CBM) muscles. The CBM originates from the apex of the coracoid process, in common with the short head of the biceps brachii muscle (BBM), and from the intermuscular septum. It inserts by means of a flat tendon into an impression at the middle of the medial surface and border of the body of the humerus, between the origins of the triceps brachii and brachialis muscles [32]. The CBM is innervated by the musculocutaneous nerve (MCN) [32].

The role of the CBM is twofold: it flexes and adducts the arm at the glenohumeral joint, and prevents the arm from being deviated from the frontal plane during abduction. Therefore, during contraction, the CBM causes shoulder flexion by drawing the humerus forward, and shoulder adduction by drawing it toward the torso. It also turns the humerus slightly inwards, thus causing internal rotation. The CBM also stabilizes the humeral head within the shoulder joint, especially when the arm hangs freely [32].

Many earlier works describe the various types of morphological variations occurring within this muscle. Some relate to accessory slips of the muscle inserting to the medial epicondyle and medial supracondylar ridge of the humerus, medial intermuscular septum of the arm, others examine additional heads or bellies, while others relate to morphological variations in the proximal or distal attachment [3, 15–17, 21, 25, 31]. Morphological variations between CBM and MCN have also been noted, namely the nerve does not pierce the muscle [10, 11, 15, 25, 47].

The presence of additional bellies for the CBM may cause musculocutaneous or high median nerve paralysis [15, 25].

This study describes a very rare case of CBM, i.e. a quadrifurcated form with a tunnel for the median nerve and MCN; it can hence act as a potential compression site for these nerves. Knowledge of such a rare type can make it easier to understand disease entities in this region and improve their treatment.

## Case report

### Morphology of the coracobrachialis muscle

**Fig. 1:**
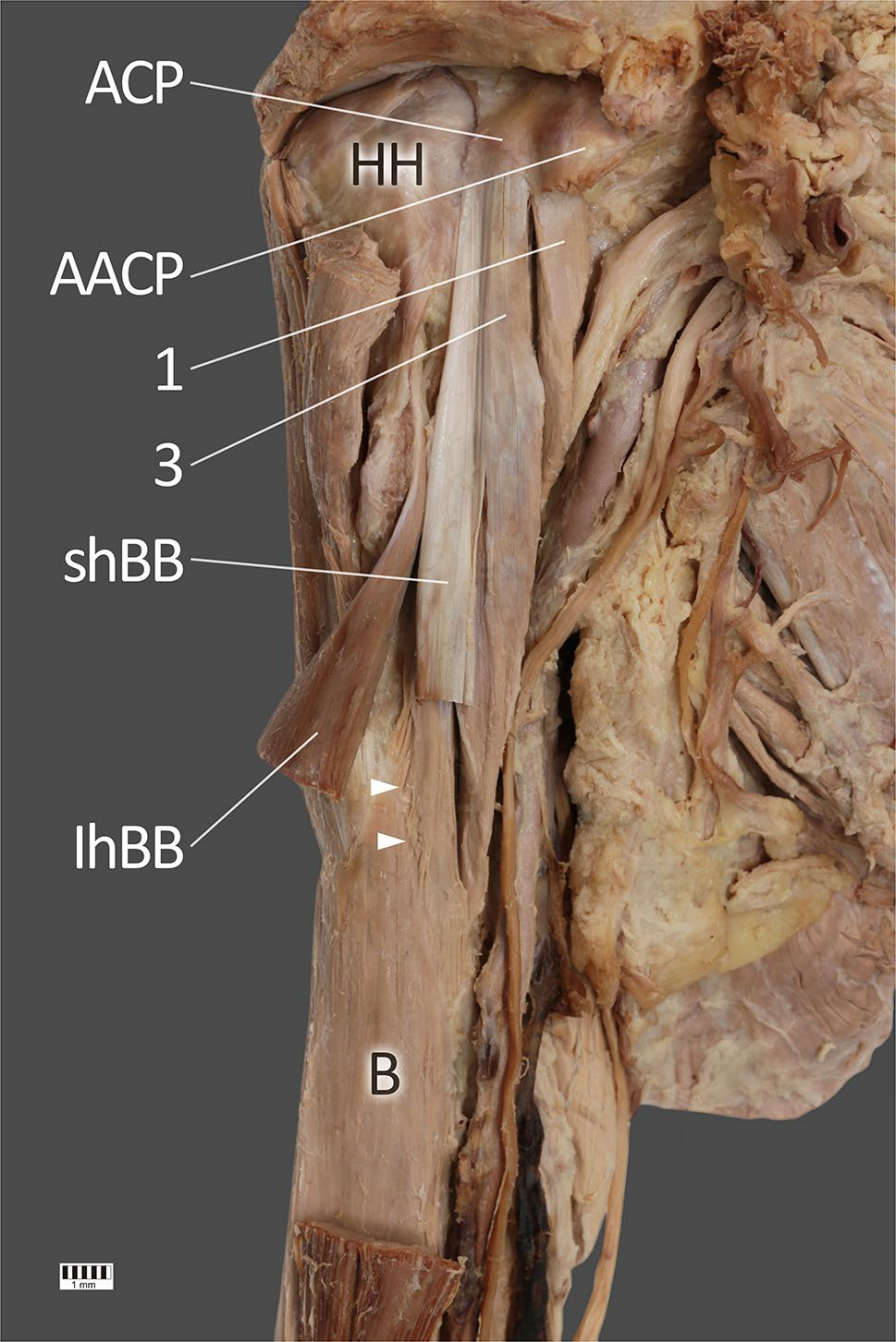
View of the three heads. Visible fusion with the short head of the biceps brachii. *HH* head of humerus, *ACP* apex of coracoid process, *AACP* accessory apex of coracoid process, *1* first head of coracobrachialis muscle, *shBB* short head of the biceps brachii, *lhBB* long head of the biceps brachii, *B* brachialis muscle. White arrowheads indicate the fusion between fourth head of the coracobrachialis muscle and brachialis muscle

**Fig. 2:**
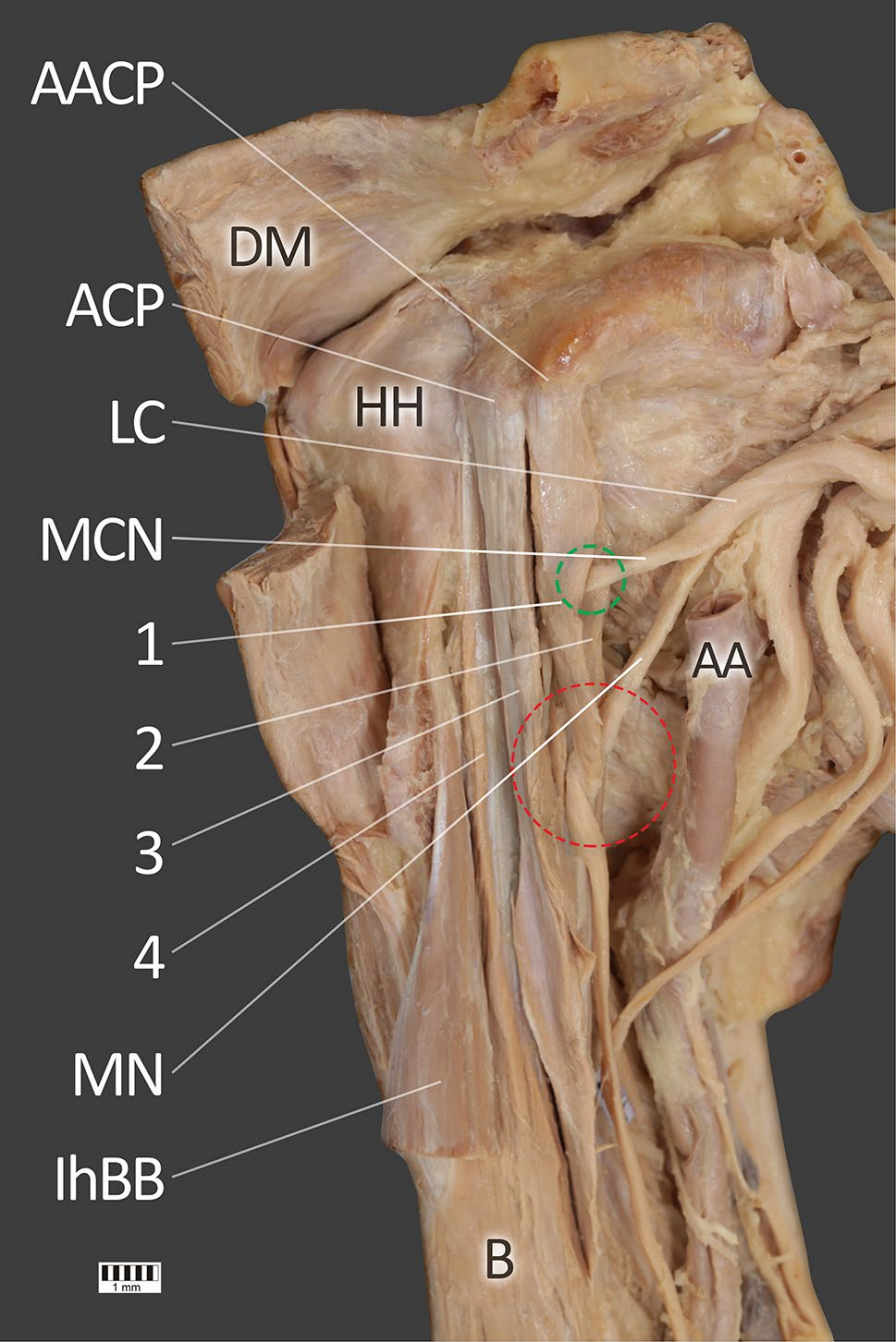
View after excision of short head of the biceps brachii. *HH* head of humerus, *ACP* apex of coracoid process, *AACP* accessory apex of coracoid process, *1* first head of coracobrachialis muscle, *2* the second head of the coracobrachialis muscle, *3* the third head of coracobrachialis muscle, *4* the fourth head of the coracobrachialis muscle, *DM* deltoid muscle, *LC* lateral cord of the brachial plexus, *MCN* musculocutaneus nerve, *MN* median nerve, *AA* axillary artery, *lhBB* long head of the biceps brachii, *B* brachialis muscle. The green circle indicates the musculocutaneus nerve ran between the first and the second head of the coracobrachialis muscle. The red circles show the place, where the median nerve ran between the first and the second head of the coracobrachialis muscle (color figure online)

**Fig. 3:**
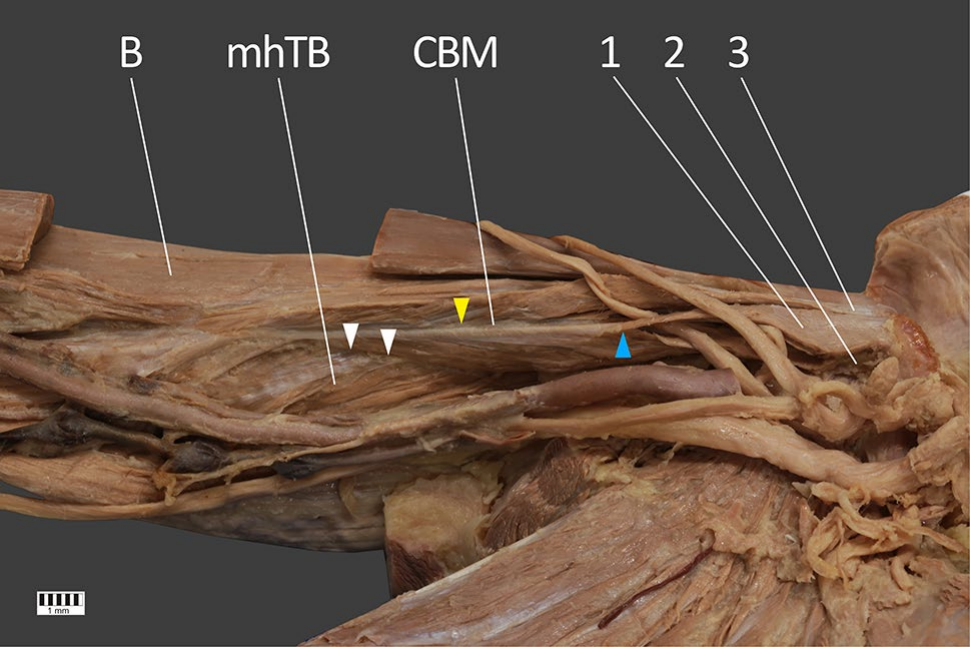
Insertion of the coracobrachialis muscle. *CBM* coracobrachialis muscle, *B* brachialis muscle, *1* first head of coracobrachialis muscle, *2* the second head of the coracobrachialis muscle, *3* the third head of coracobrachialis muscle, *mhTB* medial head of the triceps brachii. Blue arrowhead shows a connection of first and second heads of the coracobrachialis muscle. Yellow arrowhead show a connection between the third head of CBM and first and second heads joined together. White arrowheads show the connection between CBM and the medial head of the triceps brachii (color figure online)

**Fig. 4:**
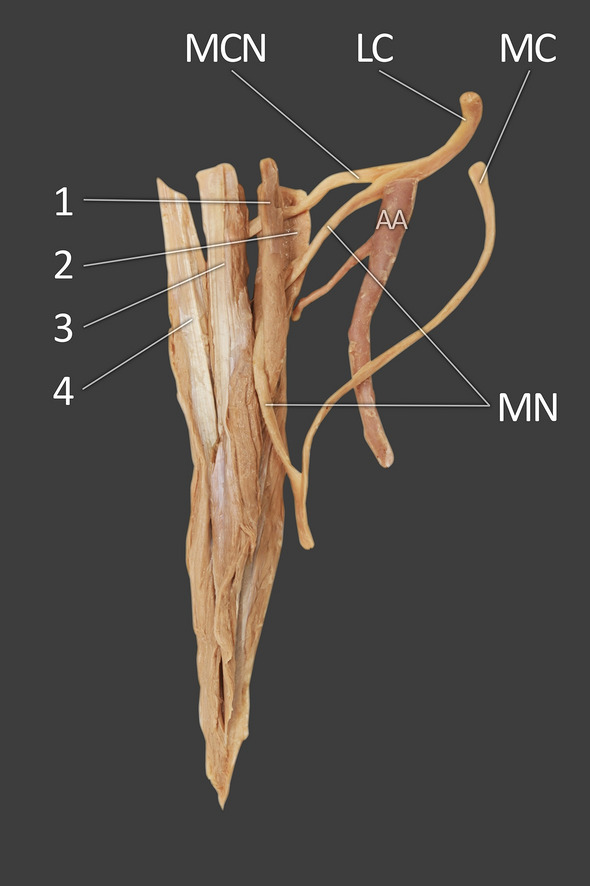
Extracted coracobrachialis muscle with nerves and arteries. *1* first head of coracobrachialis muscle, *2* the second head of the coracobrachialis muscle, *3* the third head of coracobrachialis muscle, *4* the fourth head of the coracobrachialis muscle, *MCN* musculocutaneus nerve, *MN* median nerve, *LC* lateral cord of the brachial plexus, *MC* medial cord of the brachial plexus, *AA* axillary artery

A 71-year-old female cadaver was subjected to routine anatomical dissection for research and teaching purposes in the Department of Anatomical Dissection and Donation, Medical University of Lodz, Poland. A traditional anatomical dissection of the right upper limb was performed [35–38, 41], during which, the CBM was found to be quadrifurcated—Figs. 1, 2, 3 and 4.

The next stage of the procedure involved a detailed assessment of the CBM. The CBM was characterized by four independent proximal attachments which connect with each other in the distal part; the muscle is inserted by means of a flat tendon into an impression at the middle of the medial surface and border of the humerus body. The distal part of one of the bellies was fused with the brachialis muscle—Fig. 3.

**Fig. 5:**
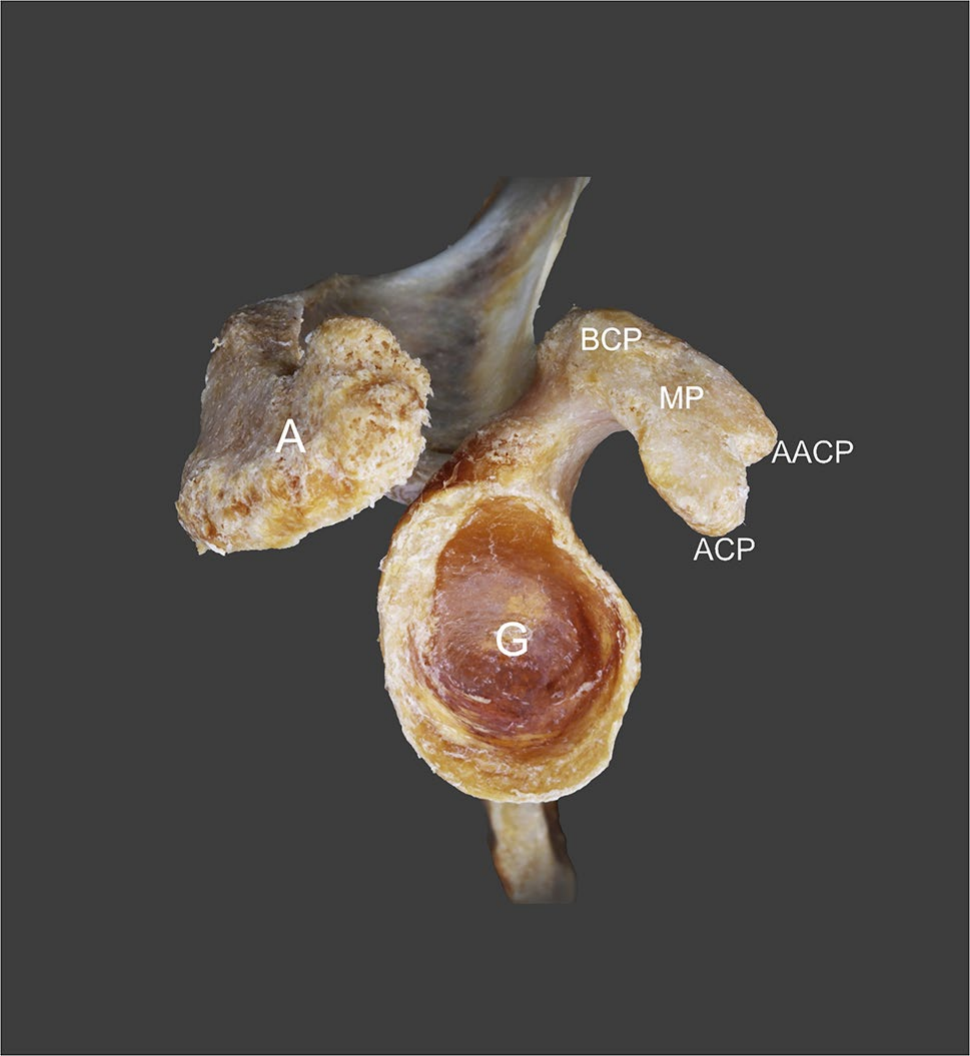
Scapula. *A* acromion, *G* glenoid fossa, *BCP* base of coracoid process, *MP* midportion, *ACP* apex of coracoid process, *AACP* accessory apex of coracoid process

**Fig. 6:**
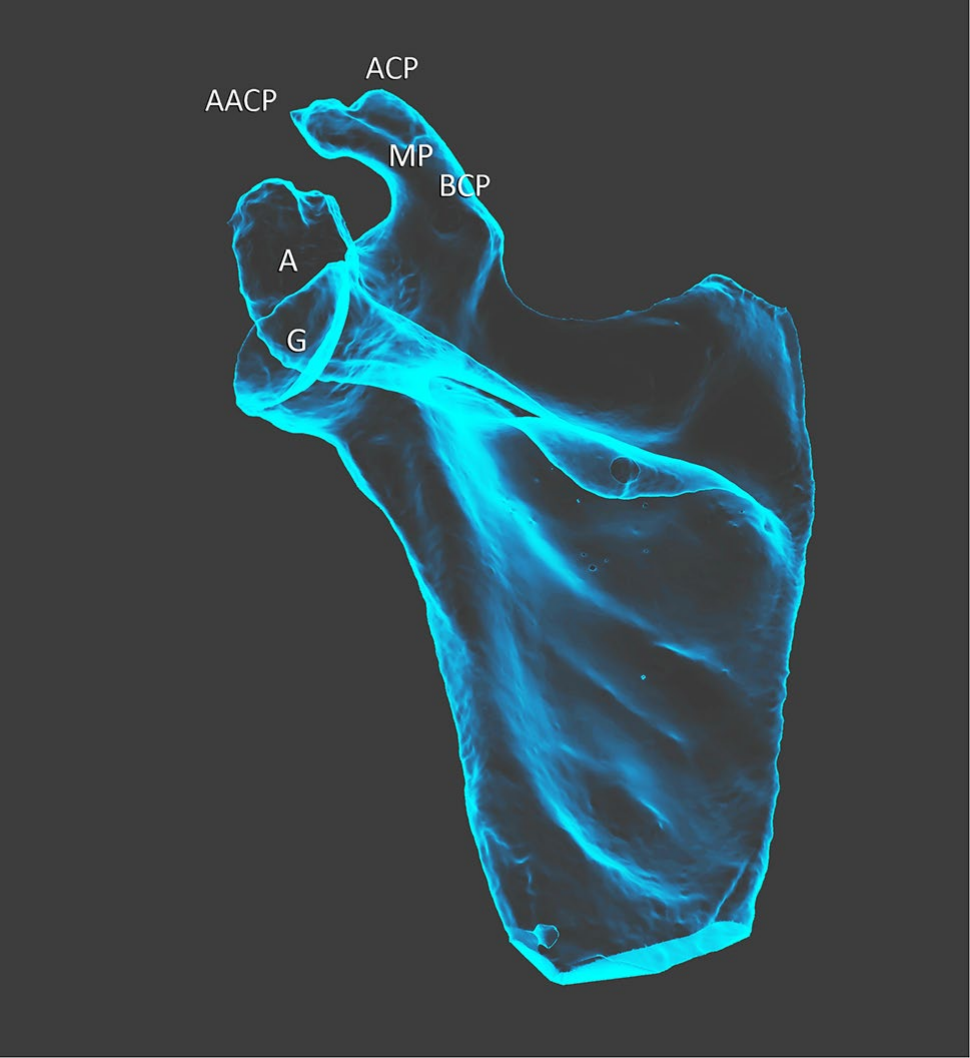
Scapula. *A* acromion, *G* glenoid fossa, *BCP* base of coracoid process, *MP* midportion, *ACP* apex of coracoid process, *AACP* accessory apex of coracoid process

**Fig. 7:**
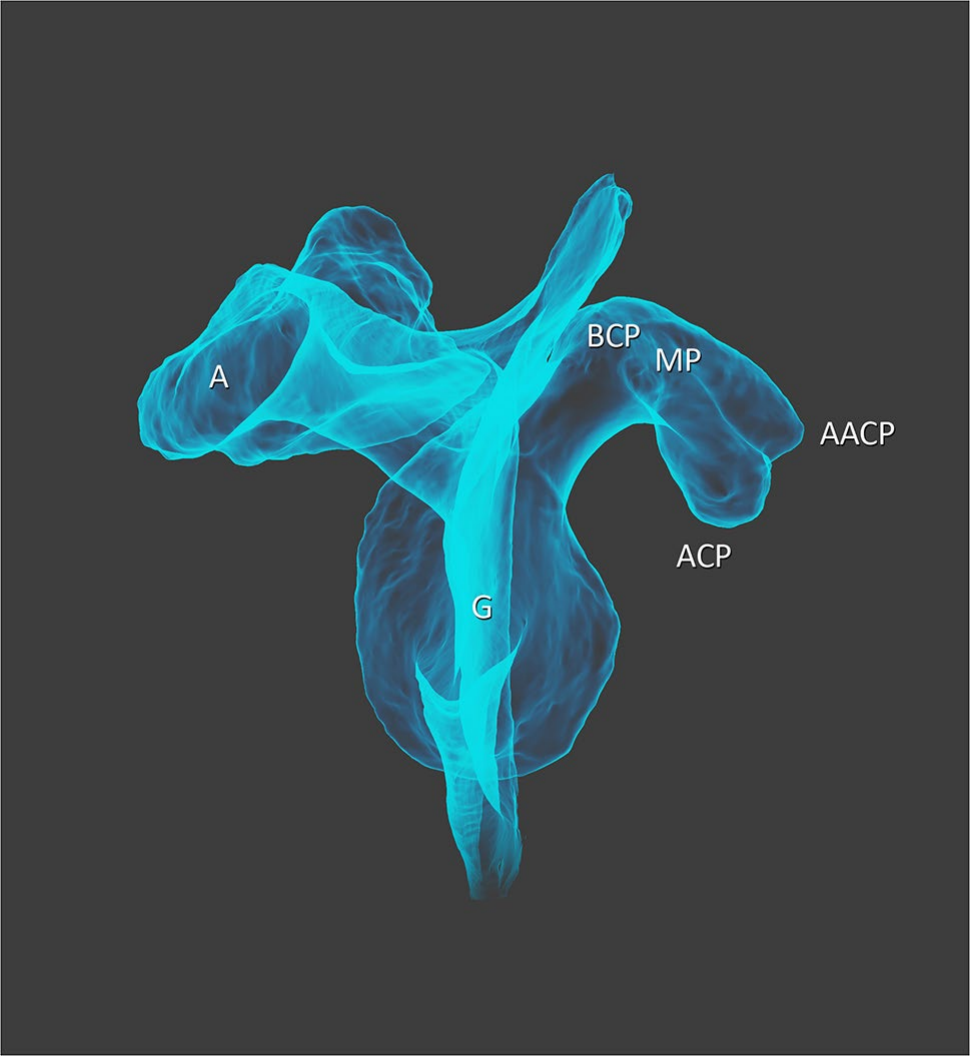
Scapula. *A* acromion, *G* glenoid fossa, *BCP* base of coracoid process, *MP* midportion, *ACP* apex of coracoid process, *AACP* accessory apex of coracoid process

**Fig. 8:**
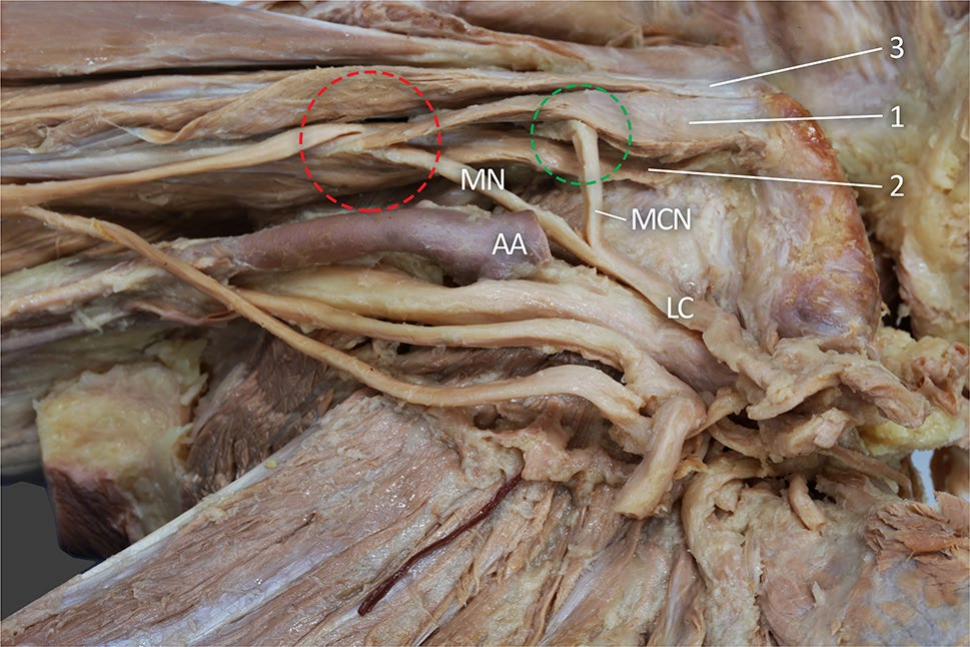
Relationship between the coracobrachialis and musculocutaneus nerve and the median nerve. *1* first head of coracobrachialis muscle, *2* the second head of the coracobrachialis muscle, *3* the third head of coracobrachialis muscle, *LC* lateral cord of the brachial plexus, *AA* axillary artery, *MCN* musculocutaneus nerve, *MN* median nerve. The green circle indicates the musculocutaneus nerve ran between the first and the second head of the coracobrachialis muscle. The red circles show the the place, where the median nerve ran between the first and the second head of the coracobrachialis muscle (color figure online)

The first two heads of the CBM demonstrate a proximal attachment at the “accessory apex” of the coracoid process of the scapula—Figs. 1, 2, 5, 6 and 7. The first, i.e. more superficial, head displaying attachments to the “accessory apex” of the coracoid process of the scapula was 77.60 mm long while the second (deeper) head, located on the inferior surface of the “accessory apex” was 71.02 mm in length. These heads closely resemble the standard attachment of this muscle; however, in this case, they represent a unilateral muscle and both the MCN and median nerve pass between the two heads—Fig. 8. The two bellies connect with each other and pass into the tendon which is 71.72 mm long—Figs. 1 and 2.

The third head, together with the head of the short biceps brachii, was attached to the apex of the coracoid process and was characterized by a fusion with the head of the short biceps brachii—Figs. 1 and 2. The length of the third head was 113.51 mm and was attached to the tendons of the first and second heads of the CBM. The fourth head was the longest (155.11 mm); it was located under the head of the short biceps brachii and displayed an attachment at the inferior surface of the coracoid process. In the distal part, it demonstrated a fusion to the brachialis muscle, with the distal attachment being at the middle of the medial surface and the border of the body of the humerus, together with the other three heads—Figs. 1 and 2.

The four heads of the CBM were innervated by the MCN—Figs. 4 and 8.

Detailed morphometric measurements were then taken. After photographic documentation, the CBM was carefully dissected to minimize any errors in measurement. The measurements were performed using two methods.

- By electronic calliper (Mitutoyo Corporation, Kawasaki-shi, Kanagawa, Japan). Each measurement was carried out twice with an accuracy of up to 0.1 mm.
- Analysis of digital photographic images processed through MultiScanBase 18.03 (Computer Scanning System II, Warsaw, Poland).

Accurate morphometric measurements can be found in Table 1.

**Table 1:** Attachment details and morphometric measurements

	Coracobrachialis muscle			
	First head	Second head	Third head	Fourth head
Origin	Accessory Apex of coracoid process of the scapula (mm)	Inferior Surface of the accessory apex of coracoid process of the scapula (mm)	Along with short head of the biceps brachii on apex of the coracoid process (mm)	Inferior surface of the coracoid process (mm)
Length of muscle belly (ventral)	77.60	71.02	113.51	155.11
Width				
Origin	10.21	9.98	3.17	2.93
Insertion	4.72			8.26
Thickness				
Origin	2.31	1.39	1.39	1.45
Insertion	3.21	1.76	1.88	2.01
Length of tendon (ventral)	71.72		38.01	8.46

### The relation of the nerves to the coracobrachialis muscle

Both MCN and the lateral root of the median nerve ran between the first and second CBM heads (muscle belly part). The mean width of the MCN passage is 3.60 mm, and that of the lateral root median nerve is 4.25 mm. The median nerve then passes between the second and third head to the anterior part of the arm; its mean width here is 4.28 mm—Fig. 8.

### Anatomical variations of the coracoid process of the scapula

The coracoid process of the scapula was characterized by a split, with two apexes visible—Figs. 1, 2, 4, 5, 6 and 7. The proximal attachments of the first and second head of the CBM had on the accessory apex, while the third and fourth heads of the CBM and the short head of the biceps brachii had the origin on the apex of the coracoid process (Table 2).

**Table 2:** Morphometric measurements

	Coracoid process	
	Standard (mm)	Accessory (mm)
Coracoid length (distance from tip to base)	50.43	42.91
Coracoid tip height	11.76	5.03
Coracoid tip width	11.59	6.20
Distance from the coracoid tip or base to the coracoid midpoint	25.21	21.45
Midpoint height	16.65	14.04
Midpoint width	15.60	15.60

## Discussion

The coracobrachialis muscle (CBM) has greater morphological than functional significance. It is, morphologically, the sole representative of the adductor group in the arm, but this functionality has been lost phylogenetically over time. The CBM has three distinct parts in amphibians, reptiles, and monotremes:

1. the coracobrachialis brevis, inserting into the humerus, superior to the latissimus dorsi tendon,
2. the coracobrachialis medius, inserted into the humerus, inferior to latissimus dorsi tendon,
3. the coracobrachialis longus or Wood’s muscle; this extends inferiorly on the shaft of humerus, where it bridges the median nerve and brachial artery.

In some primates, the CBM is composed of two parts, which is roughly equivalent to the coracobrachialis brevis. In man, it is formed of a single part; this may derive from the coracobrachialis medius of the lower animals, or from the fusion of the two heads observed in apes and prosimians; in the latter case, the MCN would be trapped between them [19, 51].

Embryologically, the variants of the CBM are believed to derive from the lateral mesoderm, together with the other muscles of the upper limb. The muscle primordia are believed to fuse to form a single body which regresses as the layers of the muscles develop. The presence of an accessory CBM could be explained as a results of the premature termination of this regression [11, 17]. The split in the scapula may arise from (a) the displacement of one of the ossification centers in utero or (b) the existence of more than two ossification centers; in this case, one of them forms a supernumerary clavicle [30, 46]. In mammals, the shoulder girdle is usually ossified from two centers, resulting in the formulation of the scapula and the coracoid process. In this case, it is possible that the ossification center for the coracoid process was single in the early period of development; it later formed a broad base that split into two from the center [30, 46].

Although the muscle commonly demonstrates morphological variations in proximal or distal attachment as well as in relation to MCN, not so much variation is observed regarding the occurrence of additional heads or bellies [8, 10, 11, 16, 17, 21, 51].

The original third head of the CBM, i.e. the coracobrachialis brevis, originates from the coracoid process and inserts into the crest of the lesser tubercle or the intertubercular groove and to the articular capsule of the shoulder joint; however, this variation is rarely seen [3–5, 8, 25, 28, 49]. The coracobrachialis brevis has also been found to attach to the shoulder joint capsule or the surgical neck of the humerus; the coracobrachialis longus might also attach to the humerus, to a fibrous band of the medial intramuscular septum, i.e. Struther’s ligament, or to the medial epicondyle [6, 50, 51]; it may also attach to the tendinous part of the latissimus dorsi [6, 51]. The CBM has been found to connect to the brachialis muscle [50], or to the brachial fascia [21]. A fetal study identified an insertion to the brachial fascia [25].

In our present study, a connection was observed between the fourth head of the CBM and the brachialis muscle (Fig. 1). A previous study described a CBM with three portions originating from the coronoid process of the scapula and inserting into the medial epicondyle of the humerus (longus), humeral diaphysis (medius) and to the humeral neck (brevis) [28]. Elsewhere another CBM variant demonstrated two bellies which formed shortly inferior to its origin from the coracoid process of the scapula: one belly inserted into the middle of the antero-medial surface of the humerus, while the other inserted into the medial head of the triceps brachii muscle [12].

Our present findings indicate the presence of a “fascial–tendinous” combination between the first, second and the third head of the CBM, and the medial head of the triceps brachii. Elsewhere, distal muscle insertions have been observed in the middle of the anteromedial humerus surface, forming an aponeurotic arch shape expansion fixed in the lateral epicondyle [11]. A three-headed CBM has also been reported previously; the first head originated from the superior border of the scapula over the scapular notch and inserted into the upper third of the medial part of the medial intramuscular septum, the second corresponded to the classical description of the CBM. Finally, the third head demonstrated proximal and distal tendinous portions and an intermediate muscle belly, the proximal tendinous part originated from the coracoid process, and inserted to the medial epicondyle of the humerus [16].

CBMs were found to be divided into superficial and deep layers in 16% of a Japanese population and incompletely divided in 8% [33]. This resembles the present case, where three of the four heads are superficial, while the fourth head is located deep under the short head of the biceps brachii. The first, second, third heads in the distal part connect to each other and attach between the brachialis muscle and the medial head of the triceps brachii. The positioning of the fourth head of CBM is very interesting. It was located under the head of the short biceps brachii and attached to the inferior surface of the apex of coracoid process; its distal part also demonstrated a fusion to the brachialis muscle, and the distal attachment was at the middle of the medial surface and border of the humerus body, together with the other three heads of CBM.

Can this fourth head represent the third head of the biceps brachii? Various types of attachment have been described for the third head of the biceps brachii, including the humeral shaft, short head of the biceps brachii and the pectoralis major [1, 26, 27]. Regarding attachment sites, in the present case, the proximal attachment was located on the scapula and the distal attachment at a site typical for CBM insertion; therefore, it appears that the fourth head is not the third head of the biceps brachii.

The CBM is believed to be functionally unimportant; however, some studies suggest that it is one of the most effective flexors of the shoulder joint and that it also resists anterior dislocation [2]. The CBM connects to the short head of the biceps brachii, and to the brachialis muscle (they are the flexors), demonstrating that it supports the pair of them. It is very interesting that the combination of three heads connects superficially with the medial head of the triceps brachii, which suggests that this structure may assist the medial head of the triceps brachii, which is an extensor; this is really interesting, because presented CBM attaches to the muscles that have an antagonistic function.

The frequency of the atypical course and relation of the MCN to CBM has been exhaustively described in the literature [10, 11, 14, 21, 29, 45, 48]. The MCN innervates the CBM in 0–22% of cases [11, 12, 14, 21, 29, 45].

A variable relationship with the surrounding nerves was observed in the present case. Both the musculoskeletal nerve and the lateral median nerve root ran between the first and second CBM heads. However, a previous studies have found the extra head of the CBM to form a “tunnel” for the median nerve and brachial artery [11] and an accessory head of the CBM which involved the lateral cord of the brachial plexus [15].

Both the CBM and accessory CBM, as well as the accessory heads of the muscle are of significant clinical importance. The CBM could be used as a guide to the axillary artery during surgery and anesthesia, and its distal attachment marks the site of the nutritional artery of the humerus [52]. The accessory head of the CBM is also of special interest in that traction on an osteomized coracoid might be expected to jeopardize not only the MCN but also a portion of the median nerve [14, 25].

The additional head identified in the present study also places pressure on both the MCN and MN, as the latter also wraps distally around the CBM. In this case, if pressure occurs, both the anterior compartment of the arm and the forearm muscle may be affected. For this reason, consideration should be given to the accessory head of the muscle before performing coracoid mobilization. MCN lesion can occur during coracoid bone block abutment (Bristow–Latarjet). The muscle inserting to the coracoid process needs to be mobilized and retracted when performing coracoid abutment transfer by Latarjet via the deltopectoral approach. This can lead to injury to the MCN, a known complication of procedures concerning the anterior shoulder. Transient lesion of the MCN may also occur following its elongation and the modification of its angle of penetration into the muscle [9]. A split coracoid process can also hinder transfer by Latarjet. In addition, other possible variations in this area should be taken into account.

Therefore, it would be advisable to perform MRI and CT of the area before planned procedures, as they play a significant role in the evaluation of anatomical and pathological lesions and anatomical variations of the shoulder and upper limb.

To summarise, surgery or interventional radiology in the shoulder and arm area should be preceded by thorough diagnostic tests to assess the presence of additional bellies or heads of the CBM. In addition, the exact course of both the MCN and MN should be assessed, together with the posterior cord to the CBM.

Morphological variations of the scapula are also quite common. These usually apply to the acromion, glenoid process and subscapular notch [20, 23, 34, 39, 40, 42–44]. In contrast, no morphological variability of the coracoid process has been observed so far; most studies concern morphometric measurements [18, 22, 24].

The present case report describes the first occurrence of a split coracoid process connected with four-headed CBM. A thorough understanding of the normal anatomy and morphological variation occurring within the acromion, coracoid process and related structures with the glenohumeral joint is necessary to correctly interpret radiological images or plan surgical procedures in the shoulder area. To avoid injury to vital neurovascular structures medial to the coracoid process, such as the brachial plexus and axillary vessels, most shoulder procedures are based on a lateral approach to the coracoid process [7, 13]. Knowledge of the anatomical variations of the coracoid process of the scapula and morphometric measurements is, therefore, highly relevant in surgical procedures involving the shoulder joint, such as hardware fixation, drill hole placement and prosthetic positioning.

## Conclusion

The CBM is characterized by great morphological variability and a variable relationship with the MCN and MN. Knowledge of these variations is necessary when planning surgical procedures in the shoulder and shoulder area, and appropriate diagnostic tests should be performed to identify additional CBM muscle heads. Knowledge of the anatomical variations of the coracoid process of the scapula and morphometric measurements is, therefore, highly relevant in surgical procedures. The presented rare case combined aforementioned muscular and bone variations and describes the first occurrence of a split coracoid process together with four-headed coracobrachialis muscle.

## Data Availability

Please contact authors for data requests (Łukasz Olewnik PhD—email address: lukasz.olewnik@umed.lodz.pl).
